# Physician preferences for prescribing Chinese patent medicines during pregnancy: a discrete choice experiment

**DOI:** 10.3389/fphar.2026.1880942

**Published:** 2026-08-03

**Authors:** Zhangyuan Yao, Yuhang Zhang, Juncheng Shu, Ting Ying, Jing Li, Yuqian Xu, Qian Zhu, Rixiang Xu, Yuanyuan Zhang, Xuefeng Xie

**Affiliations:** 1 School of Pharmacy, Anhui Medical University, Hefei, China; 2 Department of Pharmacy, The Fuyang Affiliated Hospital of Anhui Medical University, Fuyang, China; 3 School of Humanistic Medicine, Anhui Medical University, Hefei, China; 4 Key Laboratory of Public Health Social Governance, Philosophy and Social Sciences of Anhui Province, Anhui Medical University, Hefei, China; 5 Anhui Provincial Drug Regulatory Scientific Research Center, Anhui Medical University, Hefei, China

**Keywords:** Chinese patent medicines, discrete choice experiment, drug safety, pregnancy, prescribing preferences

## Abstract

**Background:**

Prescribing Chinese patent medicines (CPMs) during pregnancy requires physicians to balance fetal safety, expected maternal benefit, patient preference, affordability, and limited pregnancy-specific evidence. Quantitative evidence on how these factors are traded off remains limited.

**Objective:**

To quantify physicians’ preferences for prescribing CPMs during pregnancy, estimate the DCE-derived relative importance of key prescribing attributes, and explore preference patterns across physician subgroups.

**Methods:**

An unlabeled, forced-choice discrete choice experiment was conducted among physicians from a multi-tier medical consortium comprising 10 healthcare institutions in Anhui Province, China. Of 237 submitted questionnaires, 198 were retained for analysis, corresponding to a questionnaire validity rate of 83.54%. Seven attributes were included: fetal safety impact, treatment stage, patient willingness, prior prescribing experience with the specific CPMs, route of administration, patient affordability, and drug efficacy. A mixed logit model was used to estimate preference parameters and DCE-derived relative importance.

**Results:**

Compared with significant fetal impact, profiles indicating no fetal impact (β = 3.074, 95% CI 2.587 to 3.560, *P* < 0.001) or minor fetal impact (β = 1.954, 95% CI 1.632 to 2.276, *P* < 0.001) had higher utility. Patient willingness (β = 0.604, 95% CI 0.423 to 0.786, *P* < 0.001), topical administration (β = 0.361, 95% CI 0.143 to 0.580, *P* = 0.001), and affordability (β = 0.177, 95% CI 0.100 to 0.258, *P* < 0.001) were positively associated with profile selection. Early-pregnancy treatment (β = −0.200, 95% CI -0.397 to −0.004, *P* = 0.046) and uncertain efficacy (β = −0.316, 95% CI -0.526 to −0.107, *P* = 0.003) were negatively associated with selection. Fetal safety impact accounted for 71.07% of the total DCE-derived relative importance based on attribute-level utility ranges, followed by patient willingness and route of administration. Subgroup analyses were exploratory.

**Conclusion:**

Fetal safety was the most influential attribute in physicians’ choices between hypothetical CPMs profiles during pregnancy. Patient willingness, administration route, affordability, treatment stage, and efficacy also contributed to choices, whereas prior prescribing experience with the specific CPMs was not significantly associated with selection. The findings may inform evidence summaries, decision-support tools, and structured communication, but effects on actual prescribing and clinical outcomes require prospective evaluation.

## 1 Introduction

The safety of medication use during pregnancy is a critical concern in clinical practice and public health because medication exposure may affect both maternal and fetal outcomes (Wang R. et al., 2023). In traditional Chinese medicine (TCM), maternal and child healthcare is guided by holistic assessment and syndrome differentiation and treatment (Wang et al., 2021). Chinese patent medicines (CPMs) are standardized, industrially manufactured preparations formulated according to TCM theories and supplied in fixed dosage forms; compared with traditional decoctions, they are generally more convenient to administer and may improve patient adherence and facilitate clinical monitoring (Fang et al., 2024; Wu et al., 2020). A Chinese epidemiological survey reported that the utilization rate of Chinese patent medicines and herbal preparations among pregnant women was 52.38% (Xie, 2021; Guo et al., 2010), suggesting the clinical relevance of these medicines in maternal healthcare.

However, CPMs use during pregnancy presents particular challenges within evidence-based and integrated medical practice. A common perception that traditional or herbal medicines are natural and therefore harmless may obscure their pharmacological complexity and potential risks, contributing to irrational use during pregnancy (Wolgast et al., 2019; Nordeng et al., 2010). Studies of obstetric prescriptions have reported non-standardized CPMs use and problems related to pregnancy contraindication labeling and safety information (Guo et al., 2019; Hong et al., 2022). A study on the irrational use of medication for fetal pharmacogenetic diseases among pregnant women showed that Chinese medicines and CPMs were the main types used irrationally by pregnant women (accounting for 41.98%), and their use was associated with a significantly high incidence of fetal pharmacogenetic diseases (Huang et al., 2020). Furthermore, compared to Western pharmaceuticals, most CPMs lack high-quality, evidence-based safety classifications and clear labeling guidance for use during pregnancy, resulting in a pronounced “evidence-practice gap” (Chan, 2005; Fu et al., 2021; Shen and Tao, 2024). As a result, physicians may need to make prescribing decisions under uncertainty while simultaneously considering fetal safety, expected efficacy, treatment stage, patient willingness, route of administration, and affordability.

Understanding physicians’ decision-making logic in this complex context is crucial for advancing CPMs use from empirical practice toward standardized and individualized rational prescribing. Current research in this field follows two main paths: preclinical studies focusing on pharmacological mechanisms and embryo-fetal toxicity (Zou et al., 2024; Zhang et al., 2022; Chen et al., 2023; Di et al., 2024; Chen et al., 2017), and epidemiological analyses examining associations between drug exposure and pregnancy outcomes (Li et al., 2024; Zhu et al., 2010; Tan et al., 2024; Chan et al., 2023). Although these studies are important, they do not quantify how physicians weigh competing prescribing attributes when considering CPMs for pregnant patients. Although some studies have assessed prescribing appropriateness through prescription review or retrospective analysis, such methods cannot capture the dynamic, multidimensional preferences and choice mechanisms underlying physicians’ decisions (Guo et al., 2019; Hong et al., 2022; Xu, 2021; Shi et al., 2019). This gap in the quantitative study of decision-making limits our ability to design precise interventions to improve prescribing quality.

A discrete choice experiment (DCE) is a stated-preference method grounded in random utility theory and has been widely used to quantify trade-offs and attribute importance in healthcare decision-making (Lancsar and Louviere, 2008). By presenting respondents with hypothetical profiles that vary systematically across predefined attributes, a DCE can estimate the relative contribution of each attribute to choice and explore whether preference patterns differ across respondent subgroups. This approach is particularly suitable for studying CPMs prescribing during pregnancy because physicians may need to evaluate fetal safety, efficacy uncertainty, patient preference, cost, and route of administration jointly rather than in isolation.

Based on these clinical and methodological considerations, we expected that fetal safety, drug efficacy, and patient willingness would be important attributes in physicians’ choices between hypothetical CPMs profiles during pregnancy. Given the clinical sensitivity of pregnancy-related prescribing and the limited pregnancy-specific evidence for many CPMs, we further expected fetal safety to have relatively high importance among the included attributes. Because physicians’ experience and clinical department may influence how prescribing scenarios are interpreted, subgroup analyses by years of professional experience and clinical department were conducted as exploratory analyses.

This study aims to (1) quantify physicians’ preferences for prescribing CPMs during pregnancy, (2) estimate the relative importance of key prescribing attributes, and (3) explore preference patterns across physician subgroups. The findings are expected to provide a novel scientific perspective on clinical decision-making regarding TCM within modern healthcare systems. The findings are intended to inform the design of evidence summaries, decision-support tools, and physician-patient communication approaches, while recognizing that the DCE measures hypothetical stated preferences rather than actual prescribing behavior or clinical outcomes.

## 2 Methods

### 2.1 Study design

This study employed an unlabeled, forced-choice discrete choice experiment (DCE), grounded in random utility theory, to quantify physicians’ preferences for prescribing CPMs during pregnancy. Respondents completed paired hypothetical choice tasks defined by seven prescribing attributes and their corresponding levels. The study was conducted among physicians from a multi-tier medical consortium in Anhui Province, China.

The DCE was developed and implemented following the consensus good research practice recommendations from the International Society for Pharmacoeconomics and Outcomes Research (ISPOR) conjoint analysis task force report, including transparent attribute development, efficient experimental design, explicit coding and model specification, and reporting of uncertainty around preference estimates (Bridges et al., 2011; Reed Johnson et al., 2013; Prosser, 2016).

### 2.2 Study population

The sample size was determined following Johnson and Orme’s empirical rule for discrete choice experiments (Bekker-Grob et al., 2015). The calculation formula is N > 500c/(t × a), where c is the maximum number of attribute levels, t is the number of choice tasks per questionnaire version, and a is the number of alternatives per choice set. In this study, c = 3, t = 12, and a = 2, yielding a minimum required sample of 63 per questionnaire version. With three questionnaire versions, the overall minimum sample size was 189. To allow for incomplete or invalid online questionnaires, including incomplete responses, logical inconsistencies, rapid completion, or failure of the internal consistency check, the minimum sample size was increased by 25% and rounded up to a target sample size of 237.

Physicians were recruited from a multi-tier medical consortium in Anhui Province, China, which included the First Affiliated Hospital of Anhui Medical University and nine other institutions ranging from county-level hospitals to TCM hospitals. Eligible participants were required to: (1) hold professional authorization to independently prescribe CPMs during pregnancy, and (2) voluntarily agree to participate and ensure the authenticity of their responses in the online survey. Because the sampling frame was restricted to a single medical consortium in Anhui Province, the study was designed to characterize prescribing preferences within this consortium rather than to generate nationally representative estimates.

A stratified sampling approach was adopted based on the geographic distribution of licensed physicians in Anhui Province. Respondents were came from southern, northern, and central Anhui to ensure regional representation across the consortium’s member institutions.

### 2.3 Identification and selection of attributes and levels

The attributes and levels for the DCE were developed through a structured, stepwise process to improve clinical relevance, transparency, and reproducibility (Bridges et al., 2011; Bohorquez et al., 2024). The process included four stages: literature review and initial attribute extraction, semi-structured physician interviews, multidisciplinary expert consultation, and final confirmation of the attributes and levels.

First, a targeted literature review was conducted to identify potential factors influencing physicians’ prescribing decisions for CPMs during pregnancy. Chinese and international databases, including CNKI, Wanfang, and PubMed, were searched using terms related to “medication use during pregnancy,” “Chinese patent medicines,” “Chinese herbal medicines,” “medication safety,” “patient preference,” “physician prescribing,” and “medical decision-making.” Relevant literature on pregnancy medication use, drug safety, patient preference, and physician decision-making was reviewed (Dathe and Schaefer, 2019; Fu et al., 2021; Guo et al., 2019; Hong et al., 2022; Im et al., 2024; Shen and Tao, 2024; Hu, 2023). Methodological literature on DCE in healthcare was also consulted to ensure that the number of attributes and levels would be manageable for respondents and consistent with good practice in preference studies (Soekhai et al., 2019; Bridges et al., 2011; Bohorquez et al., 2024). Based on the literature review, potential attributes were extracted from four perspectives: medication-related factors, patient-related factors, physician-related factors, and health-system-related factors. This initial process generated eight candidate attributes: fetal safety impact, treatment stage, patient willingness, prior prescribing experience with the specific CPMs, route of administration, patient affordability, drug efficacy, and inclusion in the medical insurance catalogue.

Second, the candidate attributes and levels were refined through semi-structured interviews with 25 physicians from the Anhui medical consortium. Participants were recruited from tertiary hospitals (n = 15), secondary hospitals (n = 4), and community health centers (n = 6), covering the specialties of TCM, obstetrics, and gynecology. The interviews were conducted by trained members of the research team using a predefined interview guide. The guide focused on physicians’ real-world prescribing considerations for CPMs during pregnancy, the relevance and clarity of each candidate attribute, the appropriateness of the proposed levels, and respondents’ comprehension of the choice-task descriptions. Each interview lasted approximately 15–20 min. Interview contents were transcribed and documented in written records.

Two researchers independently reviewed the interview transcripts and written records. Content analysis was used to identify recurring prescribing considerations, unclear wording, overlapping concepts, and suggestions for revising attribute levels. Based on the interview feedback, the attribute “inclusion in the medical insurance catalogue” was removed because its practical meaning overlapped with affordability and was less directly related to physicians’ clinical trade-offs in the choice tasks. The attribute describing whether physicians considered patients’ economic status was revised as “patient affordability,” with levels defined as affordable and unaffordable.

Third, the refined attribute list was reviewed through multidisciplinary expert consultation. Seven experts were invited, including two DCE/statistical experts, three TCM researchers, and two public-health or health-policy experts. The experts evaluated the candidate attributes and levels in terms of clinical relevance, feasibility, clarity, avoidance of overlap, and suitability for DCE choice-task construction. Based on expert feedback, several attributes and levels were further revised. The attribute “drug impact on the fetus” was renamed “fetal safety impact” to more accurately reflect the prescribing concern. Treatment stage was specified as early pregnancy, mid-pregnancy, and late pregnancy. Route of administration was revised to oral, topical, and other routes. Drug efficacy was defined as good, average, and uncertain to better reflect physicians’ practical assessment of therapeutic evidence during pregnancy.

Through this literature-informed, interview-based, and expert-reviewed process, the final DCE included seven attributes. Four attributes had three levels: fetal safety impact, treatment stage, route of administration, and drug efficacy. Three attributes had two levels: patient willingness, prior prescribing experience with the specific CPM, and patient affordability. The final attributes, definitions, levels, and coding are presented in Table 1.

**TABLE 1:** Attributes and levels for DCE choice questions.

Attribute	Definition	Levels	Coding
Fetal safety impact	Degree of potential risk the medication poses to the fetus	No effect Minor effect Significant effect	Original codes: 1 = no effect; 2 = minor effect; 3 = significant effect. References for dummy coding: Significant effect
Treatment stage	Stage of pregnancy during which the CPMs would be prescribed	Early pregnancy Mid-pregnancy Late pregnancy	Original codes: 1 = early pregnancy; 2 = mid-pregnancy; 3 = late pregnancy. References for dummy coding: Late pregnancy
Patient willingness	Patient’s agreement to use the CPMs after being fully informed	Willing Unwilling	Original codes: 1 = willing; 0 = unwilling. References for dummy coding: Unwilling
Physician experience	Physician’s prior familiarity with prescribing the specific CPMs	Experienced Not experienced	Original codes: 1 = experienced; 0 = not experienced. References for dummy coding: Not experienced
Route of administration	Method by which the medication is delivered; “other” refers to non-oral and non-topical routes, such as injection, inhalation, or rectal administration	Oral Topical Other	Original codes: 1 = oral; 2 = topical; 3 = other. References for dummy coding: Other route
Patient affordability	Patient’s ability to pay the out-of-pocket cost of the medication	Affordable Unaffordable	Original codes: 1 = affordable; 0 = unaffordable. References for dummy coding: Unaffordable
Drug efficacy	Physician’s assessment of the medication’s effectiveness	Good Average Uncertain	Original codes: 1 = uncertain; 2 = average; 3 = good. References for dummy coding: Good efficacy

CPMs, Chinese Patent Medicines.

### 2.4 Questionnaire and DCE design

The survey instrument included a description of the study objectives, participant demographic characteristics, definitions of the attributes and levels, a direct rating of the perceived importance of each attribute, and the DCE choice tasks. A full factorial design would have generated 34 × 23 = 648 attribute profiles and 648 × 647/2 = 209,628 possible binary choice sets. Because a full factorial design was impractical for data collection, an efficient fractional factorial design was generated using the %ChoicEff macro in SAS, consistent with the ISPOR Conjoint Analysis Experimental Design Good Research Practices recommendations (Reed Johnson et al., 2013). The design efficiency reached a plateau at 12 experimental choice tasks per questionnaire version. Consequently, a total of 36 choice sets were generated and then randomly allocated into three separate questionnaire versions (A, B, and C) to distribute the response burden and facilitate participant completion (Reed Johnson et al., 2013).

An opt-out or no-prescription alternative was not included because the study aimed to quantify physicians’ trade-offs among alternative CPMs profiles once CPMs use was already under consideration, rather than to estimate the separate decision of whether to prescribe any CPMs. Therefore, the forced-choice design was consistent with the objective of comparing the relative influence of fetal safety, efficacy, treatment stage, patient willingness, route of administration, affordability, and prior prescribing experience across CPMs profiles. However, this design means that the DCE estimates conditional preferences between the presented profiles and does not capture physicians’ propensity to withhold CPMs treatment when neither option would be clinically acceptable. An illustrative example of a discrete choice task is provided in Table 2.

**TABLE 2:** An example of a DCE choice task.

Scene attribute	Scene 1	Scene 2
Fetal safety impact	Minor effect	No effect
Treatment stage	Mid-pregnancy	Late pregnancy
Patient willingness	Willing	Unwilling
Physician experience	Experienced	Not experienced
Route of administration	Oral	Topical
Patient affordability	Affordable	Unaffordable
Drug efficacy	Average	Good
Which scenario would you choose?	□	□

To assess response consistency, a repeated choice set was included as the final task (Choice Set 13) in each questionnaire version. One of the first 12 experimental choice sets was selected, and the order of its two alternatives was reversed. Respondents who made inconsistent choices between the original and reversed-order tasks were excluded from the analytic sample in their entirety. The repeated validation task itself was excluded from model estimation for all respondents; consequently, only the 12 experimental choice tasks contributed to the DCE analysis.

### 2.5 Data collection and statistical analysis

Data were collected using Wenjuanxing (Changsha Ranxing Information Technology Co., Ltd., China), a widely used online survey platform in China. After data collection, responses were exported and cleaned using Microsoft Excel 2023. Questionnaires were excluded if they were incomplete, contained logical inconsistencies (e.g., age lower than years of professional experience), were completed in less than 2 min, or failed the internal consistency check described above. The questionnaire validity rate was calculated as the number of questionnaires included in the analysis divided by the total number of submitted questionnaires, multiplied by 100%. The remaining data were analyzed using Stata 16.0. Descriptive statistics for categorical variables were reported as frequencies and percentages.

Conditional logit and mixed logit models were estimated within the random utility framework. Categorical attribute levels were dummy-coded, with one level of each attribute designated as the reference category and assigned a coefficient of zero. The mixed logit model was estimated by maximum simulated likelihood using 500 Halton draws. All non-reference attribute-level coefficients were specified as normally distributed random parameters, whereas the alternative-specific constant was treated as fixed. The normal distribution was selected because no *a priori* sign restriction was imposed and preferences for a given attribute level could plausibly vary in either direction across physicians. Model fit was compared using the Akaike information criterion (AIC) and Bayesian information criterion (BIC), with lower values indicating better fit. Based on these criteria, the mixed logit model was selected as the final model for estimating preference parameters, calculating the relative importance of attributes, and exploring preference heterogeneity across physician subgroups (Wang L. et al., 2023; Prosser, 2016).

The original categorical attribute levels were numerically coded as shown in Table 1. Before model estimation, these categorical variables were converted into dummy variables, with one level of each attribute designated as the reference category and assigned a coefficient of zero. The final mixed logit model included 11 non-reference dummy variables representing fetal safety impact, treatment stage, patient willingness, prior prescribing experience with the specific CPMs, route of administration, patient affordability, and drug efficacy.

Model estimation was conducted in Stata 16.0 using clogit for the conditional logit model and the user-written mixlogit command for the mixed logit model. The data were organized in long format, with select denoting the chosen alternative, grip identifying each choice set, and person_id identifying each respondent. Detailed variable coding and Stata command structures are provided in Supplementary Material.

Relative importance scores were computed based on the mean preference coefficients estimated from the mixed logit model. For each attribute, its utility range was defined as the difference between the maximum and minimum coefficients across all corresponding levels, incorporating the reference level fixed at a coefficient of zero (Prosser, 2016). The relative importance of attribute k was calculated as:

$$RI_{k}=\frac{\max\left(\beta_{k}\right)-\min\left(\beta_{k}\right)}{\sum_{j}\left[\max\left(\beta_{j}\right)-\min\left(\beta_{j}\right)\right]}\times 100\%$$

where the denominator represents the total sum of utility ranges across all seven experimental attributes. Directly rated importance percentages were calculated by dividing the mean rating for each attribute by the sum of the mean ratings across all attributes and multiplying by 100%.

Exploratory subgroup mixed logit models were estimated separately according to years of professional experience (≤10 years vs. >10 years) and clinical department (Department of Traditional Chinese Medicine vs. Department of Obstetrics and Gynecology). No formal interaction tests were performed; therefore, subgroup-specific coefficients were interpreted descriptively and were not considered evidence of statistically confirmed between-group differences. Results are reported as beta coefficients (β), standard errors (SEs), two-sided P values, and 95% confidence intervals (CIs). A two-sided P value < 0.05 was considered statistically significant.

## 3 Results

### 3.1 Demographic characteristics

After excluding questionnaires that were incomplete, contained logical inconsistencies, were completed in less than 2 min, or failed the internal consistency check, 198 of 237 submitted questionnaires were retained for analysis, corresponding to a questionnaire validity rate of 83.54%. The analytic sample comprised physicians from southern (n = 32), central (n = 106), and northern (n = 60) Anhui. Participants were predominantly female (155/198, 78.28%) and had a Western medicine training background (144/198, 72.73%). The mean age was 38.51 years, and the mean duration of professional practice was 10.88 years. The largest age group was 31–40 years (n = 106, 53.54%). Fifty-three physicians (26.77%) worked in departments of TCM, and 145 (73.23%) worked in departments of obstetrics and gynecology. Detailed demographic characteristics are presented in Table 3.

**TABLE 3:** Demographic characteristics.

Demographic characteristics	N (%)
Work location	
Southern anhui	32 (16.16)
Northern anhui	60 (30.30)
Central anhui	106 (53.54)
Gender	
Male	43 (21.72)
Female	155 (78.28)
Years of working	
1–10	87 (43.94)
11–20	71 (35.86)
21–30	24 (12.12)
≥30	16 (8.08)
Age	
21–30	25 (12.63)
31–40	106 (53.54)
41–50	47 (23.74)
≥51	20 (10.10)
Medical training background	
Western medicine	144 (72.73)
Traditional Chinese medicine	40 (20.20)
Integrated Chinese and Western medicine	14 (7.07)
Degree of education	
Technical secondary school	1 (0.51)
Junior college	10 (5.05)
Undergraduate course	105 (53.03)
Master degree candidate	62 (31.31)
Doctoral candidate	20 (10.10)
Professional title	
Assistant physician	11 (5.56)
Physician	32 (16.16)
Visiting staff	85 (42.93)
Associate chief physician	52 (26.26)
Chief physician	18 (9.09)
Classification of medical practitioner certificates	
Medical practitioner	195 (98.48)
Assistant medical practitioner	3 (1.52)
Clinical department	
Department of traditional Chinese medicine	53 (26.77)
Department of obstetrics and gynecology	145 (73.23)
Work unit	
Class a tertiary hospital	141 (71.21)
Class B secondary hospital	52 (26.26)
Primary hospital	5 (2.52)

CPMs, Chinese Patent Medicines.

### 3.2 Physicians’ prescribing preferences for CPMs

To analyze physicians’ prescribing preferences, both conditional logit and mixed logit models were estimated. Model comparison based on the Akaike information criterion (AIC) and Bayesian information criterion (BIC) indicated that the mixed logit model (AIC = 2,223.136, BIC = 2,358.920) provided a better fit than the conditional logit model (AIC = 2,431.443, BIC = 2,496.102). Consequently, the mixed logit model was selected for the primary analysis.

Results from the mixed logit model are presented in Table 4. Several attribute levels were significantly associated with profile selection. Compared with profiles indicating significant fetal impact, profiles indicating no fetal impact (β = 3.074, 95% CI 2.587 to 3.560, *P* < 0.001) or minor fetal impact (β = 1.954, 95% CI 1.632 to 2.276, *P* < 0.001) had substantially higher utility. Profiles were also more likely to be selected when the patient was willing to use CPMs after being fully informed (β = 0.604, 95% CI 0.423 to 0.786, *P* < 0.001), when administration was topical rather than through another route (β = 0.361, 95% CI 0.143 to 0.580, *P* = 0.001), and when the treatment was affordable to the patient (β = 0.177, 95% CI 0.100 to 0.258, *P* < 0.001). Early-pregnancy treatment was associated with lower utility than late-pregnancy treatment (β = −0.200, 95% CI -0.397 to −0.004, *P* = 0.046), as was uncertain rather than good efficacy (β = −0.316, 95% CI -0.526 to −0.107, *P* = 0.003). Oral administration (β = 0.201, *P* = 0.059), mid-pregnancy treatment (β = 0.037, *P* = 0.690), and average efficacy (β = 0.081, *P* = 0.444) were not statistically significant.

**TABLE 4:** Mixed-logit model estimates (n = 198).

Attribute and level	β (SE)	*P*	95% CI for β	SD (SE)	*P*	95% CI for SD
Fetal safety impact (ref: significant effect)						
Minor effect	1.954 (0.164)	<0.001	1.632–2.276	2.279 (0.249)	<0.001	1.791–2.767
No effect	3.074 (0.248)	<0.001	2.587–3.560	1.043 (0.153)	<0.001	0.743–1.342
Treatment stage (ref: late pregnancy)						
Mid-pregnancy	0.037 (0.094)	0.690	−0.146–0.221	−0.018 (0.207)	0.931	−0.424–0.387
Early pregnancy	−0.200 (0.100)	0.046	−0.397–−0.004	0.303 (0.210)	0.149	−0.110–0.714
Patient willingness (ref: unwilling)						
Willing	0.604 (0.093)	<0.001	0.423–0.786	0.708 (0.114)	<0.001	0.485–0.931
Physician experience (ref: not experienced)						
Experienced	0.069 (0.072)	0.337	−0.072–0.209	−0.086 (0.179)	0.630	−0.436–0.264
Route of administration (ref: other)						
Topical	0.361 (0.111)	0.001	0.143–0.580	0.312 (0.206)	0.129	−0.091–0.714
Oral	0.201 (0.106)	0.059	−0.007–0.408	0.313 (0.177)	0.077	−0.034–0.660
Patient affordability (ref: unaffordable)						
Affordable	0.177 (0.041)	<0.001	0.100–0.258	0.0007 (0.062)	0.990	−0.121–0.122
Drug efficacy (ref: good)						
Average	0.081 (0.106)	0.444	−0.126–0.288	0.122 (0.181)	0.500	−0.232–0.476
Uncertain	−0.316 (0.107)	0.003	−0.526–−0.107	−0.496 (0.166)	0.003	−0.821–−0.170

β: Beta Coefficient, SE: standard error, *P*:*P*-value, CI: confidence interval, SD: standard deviation.

Prior prescribing experience with the specific CPMs was not associated with profile selection (β = 0.069, 95% CI -0.072 to 0.209, *P* = 0.337), and its random-parameter standard deviation was also not statistically significant (SD = −0.086, *P* = 0.630). Significant random-parameter standard deviations were observed for minor fetal impact, no fetal impact, patient willingness, and uncertain efficacy (all *P* ≤ 0.003), indicating heterogeneity in preferences for these attribute levels. The standard deviations for treatment stage, route of administration, patient affordability, average efficacy, and prior prescribing experience were not statistically significant.

### 3.3 Relative importance of attributes

**FIGURE 1:**
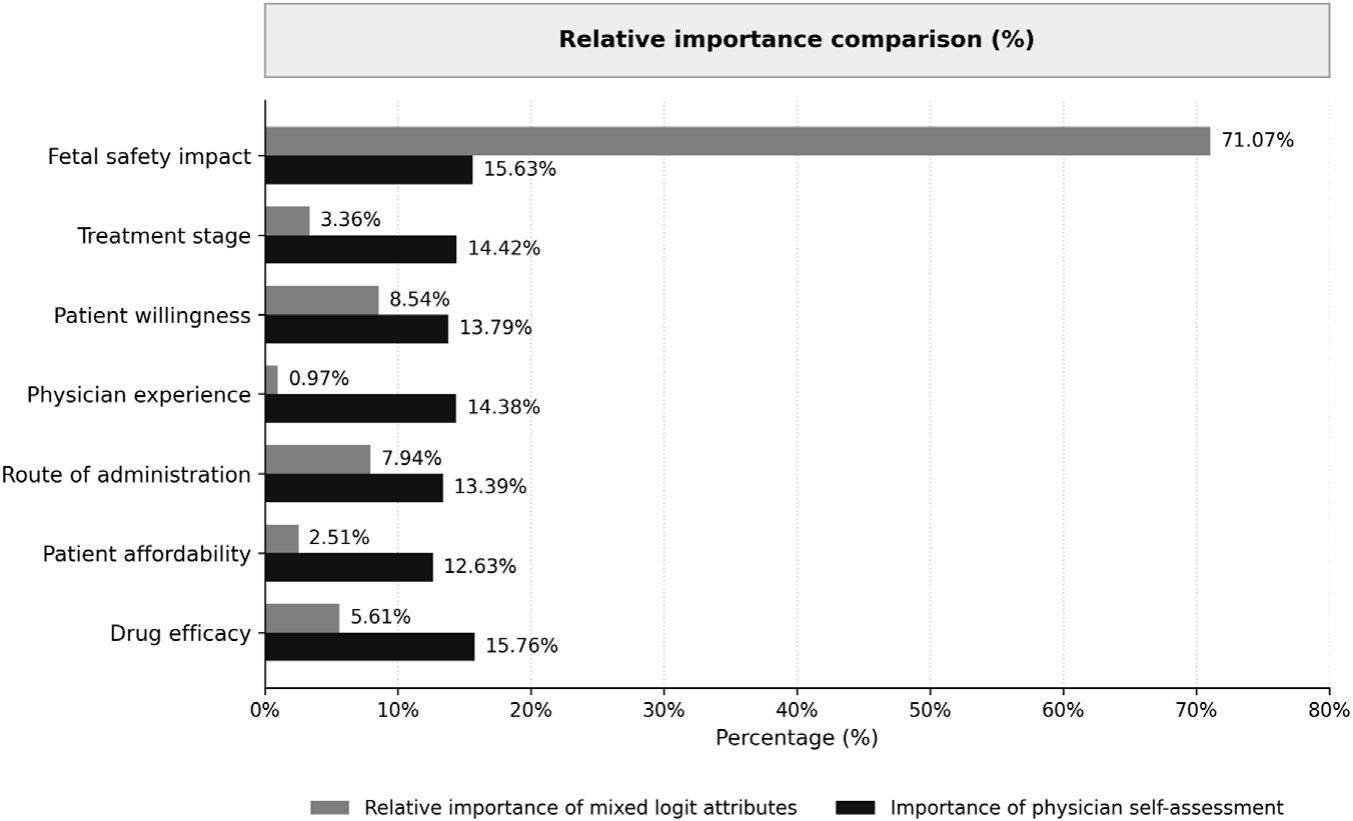
Percentage of importance of inclusion attributes.

A substantial descriptive difference was observed between directly rated attribute importance and DCE-derived choice-based relative importance. In the direct rating exercise, the percentages were relatively evenly distributed across drug efficacy (15.76%), fetal safety impact (15.63%), treatment stage (14.42%), prior prescribing experience with the specific CPMs (14.38%), patient willingness (13.79%), route of administration (13.39%), and patient affordability (12.63%). In contrast, based on the utility ranges estimated by the mixed logit model, fetal safety impact accounted for 71.07% of the total DCE-derived relative importance, followed by patient willingness (8.54%), route of administration (7.94%), drug efficacy (5.61%), treatment stage (3.36%), patient affordability (2.51%), and prior prescribing experience (0.97%) (Figure 1).

The largest descriptive differences between the two measurement approaches concerned fetal safety impact and prior prescribing experience. Fetal safety impact ranked second in the direct rating exercise but first in the DCE-derived measure, whereas prior prescribing experience ranked fourth in the direct ratings but last in the DCE-derived measure. These results indicate that the ranking of attributes differed according to whether importance was assessed directly or inferred from choices among hypothetical profiles.

### 3.4 Subgroup analysis

Exploratory subgroup mixed logit results by clinical department and years of professional experience are presented in Tables 5, 6, respectively. Because the models were estimated separately and no formal interaction tests were performed, differences in coefficient magnitude or statistical significance between subgroups were interpreted descriptively and do not establish between-group differences. In both the TCM department subgroup (n = 53) and the obstetrics and gynecology subgroup (n = 145), profiles indicating minor or no fetal impact, patient willingness, topical administration, and patient affordability were positively associated with selection, whereas prior prescribing experience was not statistically significant. Within the obstetrics and gynecology subgroup, early-pregnancy treatment was negatively associated with selection (β = −0.233, 95% CI -0.448 to −0.019, *P* = 0.033), as was uncertain efficacy (β = −0.530, 95% CI -0.767 to −0.292, *P* < 0.001). The corresponding coefficients were not statistically significant within the TCM department subgroup. In that subgroup, average efficacy was positively associated with selection relative to good efficacy (β = 0.422, 95% CI 0.002 to 0.841, *P* = 0.049).

**TABLE 5:** Sub-group mixed-logit analysis: clinical department.

Attributes and levels	Traditional Chinese medicine				Obstetrics and gynecology			
	β	SE	*P*	95% CI	β	SE	*P*	95% CI
Fetal safety impact (ref: significant effect)								
Minor effect	1.567	0.307	<0.001	0.966–2.168	1.978	0.149	<0.001	1.686–2.270
No effect	2.555	0.512	<0.001	1.553–3.559	3.016	0.230	<0.001	2.564–3.467
Treatment stage (ref: late pregnancy)								
Mid-pregnancy	−0.099	0.222	0.656	−0.534–0.336	0.083	0.109	0.445	−0.130–0.297
Early pregnancy	−0.026	0.205	0.899	−0.437–0.375	−0.233	0.109	0.033	−0.448–−0.019
Patient willingness (ref: unwilling)								
Willing	0.611	0.163	<0.001	0.291–0.930	0.588	0.101	<0.001	0.390–0.787
Physician experience (ref: not experienced)								
Experienced	0.204	0.148	0.166	−0.085–0.493	0.050	0.084	0.548	−0.114–0.214
Route of administration (ref: other)								
Topical	0.441	0.218	0.043	0.014–0.868	0.367	0.134	0.006	0.105–0.629
Oral	0.294	0.198	0.138	−0.095–0.683	0.183	0.119	0.124	−0.050–0.416
Patient affordability (ref: unaffordable)								
Affordable	0.161	0.080	0.043	0.005–0.317	0.183	0.048	<0.001	0.089–0.278
Drug efficacy (ref: good)								
Average	0.422	0.214	0.049	0.002–0.841	−0.219	0.120	0.067	−0.454–0.015
Uncertain	0.199	0.203	0.327	−0.198–0.596	−0.530	0.121	<0.001	−0.767–−0.292

β, Beta Coefficient; SE, standard error; *P*, *P*-value; CI, confidence interval.

**TABLE 6:** Sub-group mixed-logit analysis: years of working.

Attributes and levels	≤10 years				>10 years			
	β	SE	*P*	95% CI	β	SE	*P*	95% CI
Fetal safety impact (ref: significant effect)								
Minor effect	1.970	0.250	<0.001	1.480–2.461	1.798	0.165	<0.001	1.475–2.122
No effect	3.407	0.394	<0.001	2.634–4.180	2.773	0.282	<0.001	2.220–3.327
Treatment stage (ref: late pregnancy)								
Mid-pregnancy	0.286	0.167	0.086	−0.040–0.613	−0.086	0.113	0.442	−0.307–0.134
Early pregnancy	0.180	0.170	0.289	−0.153–0.512	−0.324	0.115	0.005	−0.550–−0.099
Patient willingness (ref: unwilling)								
Willing	0.612	0.144	<0.001	0.329–0.895	0.613	0.106	<0.001	0.404–0.820
Physician experience (ref: not experienced)								
Experienced	0.009	0.124	0.943	−0.235–0.252	0.109	0.084	0.198	−0.057–0.274
Route of administration (ref: other)								
Topical	0.454	0.185	0.014	0.091–0.817	0.388	0.127	0.002	0.138–0.638
Oral	0.174	0.183	0.341	−0.184–0.532	0.244	0.123	0.047	0.004–0.484
Patient affordability (ref: unaffordable)								
Affordable	0.165	0.070	0.019	0.027–0.303	0.184	0.051	<0.001	0.084–0.284
Drug efficacy (ref: good)								
Average	−0.324	0.181	0.073	−0.678–0.030	−0.007	0.124	0.952	−0.250–0.235
Uncertain	−0.422	0.174	0.015	−0.763–−0.080	−0.276	0.105	0.008	−0.482–−0.071

β, Beta Coefficient; SE, standard error; *P*, *P*-value; CI, confidence interval.

When stratified by years of professional experience (≤10 years vs. >10 years), profiles indicating minor or no fetal impact, patient willingness, topical administration, and patient affordability were positively associated with selection in both subgroups, whereas uncertain efficacy was negatively associated with selection in both subgroups (Table 6). Within the >10-year subgroup, early-pregnancy treatment was negatively associated with profile selection (β = −0.324, 95% CI -0.550 to −0.099, *P* = 0.005), whereas the corresponding coefficient was not statistically significant in the ≤10-year subgroup (β = 0.180, 95% CI -0.153 to 0.512, *P* = 0.289). Oral administration was positively associated with selection only within the >10-year subgroup (β = 0.244, 95% CI 0.004 to 0.484, *P* = 0.047). Prior prescribing experience was not statistically significant in either subgroup. These subgroup-specific results are descriptive and should not be interpreted as formal evidence of differences by years of professional experience.

## 4 Discussion

This study used a forced-choice discrete choice experiment to examine how physicians within a medical consortium in Anhui Province traded off attributes of CPMs profiles for use during pregnancy. The mixed logit results showed that fetal safety had the largest DCE-derived relative importance, while patient willingness, route of administration, drug efficacy, treatment stage, and affordability also contributed to profile selection. Prior prescribing experience with the specific CPMs was not significantly associated with profile selection. The direct rating exercise and the DCE-derived measure produced different rankings, and exploratory subgroup models showed broadly consistent core preferences across clinical departments and experience groups.

Fetal safety accounted for 71.07% of the total DCE-derived relative importance based on attribute-level utility ranges. This percentage should be interpreted as a relative measure conditional on the attributes and levels included in the experiment, rather than as the proportion of preference variance explained or as an absolute measure of clinical importance. In this study, fetal safety referred to the potential impact of medication exposure on the fetus, encompassing teratogenicity as well as other possible consequences, such as fetotoxicity, impaired fetal growth or functional development, and adverse neonatal effects (Dathe and Schaefer, 2019). Because the questionnaire did not ask respondents to distinguish among these specific outcomes, the findings should be interpreted as reflecting a general concern about fetal safety rather than any single type of fetal harm. The strong preference for profiles with no or minor fetal impact is consistent with the central role of fetal risk assessment in medication use during pregnancy (Feng and Gu, 2002; Honein et al., 2013). The difference between the direct ratings and the DCE-derived rankings should not be interpreted as evidence of a subconscious preference. Direct ratings assess attributes separately, whereas DCE tasks require respondents to trade attributes off within complete profiles; direct responses may also be affected by scale-use patterns or social desirability. Differences between elicitation methods have been reported in preference research (Veldwijk et al., 2024).

The overall model also indicated lower utility for early-pregnancy treatment than for late-pregnancy treatment and lower utility for uncertain rather than good efficacy. Caution regarding treatment during early pregnancy is clinically plausible because this period includes critical stages of fetal development, but the DCE did not directly measure physicians’ reasons for assigning lower utility to early-pregnancy profiles. Similarly, the negative coefficient for uncertain efficacy suggests that physicians considered therapeutic evidence alongside fetal safety rather than focusing on risk alone. The limited pregnancy-specific evidence and incomplete labeling for many CPMs may make both benefit and risk difficult to assess (Fu et al., 2021; Shen and Tao, 2024). Therefore, the results support the need to present safety, efficacy, and uncertainty together rather than assuming a simple shift from an efficacy-first to a safety-first decision rule.

Patient willingness was the second most important attribute in the DCE-derived analysis, and affordability had a smaller but statistically significant positive association with profile selection. These findings are compatible with the relevance of patient autonomy, informed communication, treatment acceptability, and financial feasibility in medication decisions (Chen et al., 2020; Hu, 2023; Im et al., 2024). Nevertheless, patient willingness should not be interpreted as an independent clinical indication or as transferring responsibility for risk to the patient. Instead, it highlights the importance of explaining available evidence, uncertainty, alternatives, and potential maternal and fetal consequences before eliciting patient preferences. The affordability result likewise indicates that physicians considered patients’ ability to bear out-of-pocket costs, but the study did not evaluate cost-effectiveness, insurance policy, or actual access to specific CPMs.

Topical administration was preferred to the reference category of other routes, whereas oral administration did not reach statistical significance in the overall model. One possible explanation is that physicians may perceive topical treatment as producing more localized exposure and less systemic absorption. However, the survey did not measure these beliefs directly, and topical administration cannot be assumed to be uniformly safer: systemic exposure depends on the active ingredients, formulation, dose, application area, duration, and integrity of the skin or mucosa. The route finding should therefore be interpreted as a stated preference within the experimental profiles rather than evidence that topical CPMs are clinically safer during pregnancy (Patel et al., 2019).

An important null finding was that prior prescribing experience with the specific CPMs was not significantly associated with profile selection, either in the overall model or in the retained subgroup models. This contrasts with its relatively high position in the direct rating exercise and suggests that familiarity contributed little additional utility when respondents were required to trade it off against fetal safety, efficacy, patient willingness, route, timing, and affordability. This result does not demonstrate that clinical experience is irrelevant to prescribing; rather, it indicates that the specific experience attribute, as defined in this DCE, was not independently associated with choices. Significant random-parameter standard deviations for fetal safety, patient willingness, and uncertain efficacy further indicate that physicians were not homogeneous in their preferences for these levels.

The subgroup analyses should be interpreted as exploratory. Across both clinical-department subgroups and both professional-experience subgroups, profiles with less fetal impact, patient willingness, topical administration, and affordability generally had positive coefficients, while prior prescribing experience was not statistically significant. Within the obstetrics and gynecology subgroup and the group with at least 10 years of professional experience, early-pregnancy treatment was negatively associated with profile selection; uncertain efficacy was also negatively associated with selection within the obstetrics and gynecology subgroup. However, the models were estimated separately and no formal interaction tests were performed. Consequently, differences in coefficient magnitude or statistical significance cannot establish that preferences differed between departments or experience groups. The positive coefficient for average efficacy in the smaller TCM department subgroup was borderline in statistical significance and should not be given a substantive interpretation without replication.

The findings may inform the content and priorities of future clinical guidance, decision-support tools, and educational interventions, but they do not demonstrate that any particular intervention will improve prescribing or pregnancy outcomes. To make the translation from descriptive preference estimates to candidate intervention components explicit, each main finding can be linked to a potential design priority. First, because fetal safety had the highest DCE-derived relative importance, a pregnancy-specific CPMs decision-support interface could place a concise summary of the type of potential fetal harm, evidence quality, and degree of uncertainty at the beginning of the assessment rather than presenting safety as an undifferentiated warning. Second, the lower utility assigned to early-pregnancy profiles supports organizing information by gestational stage and prompting clinicians to document gestational age, the maternal indication, expected benefit, and risks of delaying or withholding treatment. Third, because uncertain efficacy reduced profile utility, safety evidence should be paired with information on expected maternal benefit, efficacy uncertainty, available alternatives, and the consequences of no treatment. Fourth, the findings for route of administration and affordability suggest that a tool could prompt consideration of route-specific exposure and practical access without assuming that topical administration is inherently safer. Finally, because prior familiarity with the specific CPMs was not independently associated with profile selection, decision support and physician training should emphasize retrieval and critical appraisal of current pregnancy-specific evidence rather than reliance on habitual familiarity alone. These proposed components are design hypotheses derived from the observed preference structure, not prescriptive clinical rules. General evidence suggests that clinical decision-support systems can assist medication prescribing, although their effectiveness depends on design and implementation (Armando et al., 2023). Because formal subgroup comparisons were not performed, the present findings do not support mandatory department-specific or experience-specific protocols; any tailored training strategy requires confirmation in adequately powered studies.

The prominence of patient willingness also provides a specific rationale for structured physician-patient communication. A practical framework could prompt clinicians to explain the maternal indication, potential fetal and maternal benefits and harms, uncertainty in the evidence, available alternatives, route of administration, and financial burden, and then document the patient’s values and preferences. Such a framework would support informed shared decision-making rather than merely seeking agreement to use a CPMs. Decision aids in obstetrics and gynecology have been associated with improved knowledge and reduced decisional conflict, but a CPMs-specific tool would require co-design, usability testing, and prospective evaluation before clinical implementation.

This study has several limitations that suggest directions for future research. First, the sample was drawn from a single province (Anhui) within a medical consortium, which may limit the generalizability of findings to other regions, particularly those with different economic development levels or healthcare infrastructures. Second, the forced-choice design did not include an opt-out or no-prescription alternative. Therefore, the study estimated trade-offs conditional on choosing between two CPMs profiles and may have required respondents to select a profile even when neither option would have been acceptable in real clinical practice. The results should therefore not be interpreted as physicians’ absolute willingness to prescribe CPMs during pregnancy. Third, although attributes were developed through physician interviews and expert consultation, no DCE can include every determinant of prescribing (Bridges et al., 2011; Bohorquez et al., 2024). Potentially relevant factors not represented included maternal disease severity and urgency, expected maternal benefit, risks of no treatment, and clinical guidelines, alongside numerous other contextual and institutional considerations. Fourth, online recruitment and questionnaire exclusions may have introduced selection bias. Fifth, the study measured stated preferences in hypothetical tasks rather than actual prescriptions or maternal and fetal outcomes. Finally, the subgroup analyses were exploratory. Separate mixed logit models were estimated by years of professional experience and clinical department, but formal interaction tests were not performed. Therefore, differences in coefficient magnitude or statistical significance across subgroup-specific models should not be interpreted as definitive evidence of between-group heterogeneity.

Future research should replicate the study in larger and more geographically and institutionally diverse physician samples. Future DCEs could incorporate a no-prescription option and additional attributes such as maternal disease severity, treatment alternatives, institutional constraints, and legal or guideline considerations. Linking preference data with real-world prescriptions and subsequent maternal and fetal outcomes would help assess the preference-behavior-outcome relationship. Finally, decision aids, prescribing support systems, and communication or training interventions developed from these findings should be co-designed with physicians and pregnant patients and evaluated in prospective implementation studies before claims of improved prescribing safety are made.

## 5 Conclusion

In this forced-choice DCE among 198 physicians from one medical consortium in Anhui Province, fetal safety had the highest DCE-derived relative importance in choices between hypothetical CPMs profiles for use during pregnancy. Patient willingness, topical administration, affordability, treatment stage, and efficacy also contributed to profile selection, whereas prior prescribing experience with the specific CPMs was not significantly associated with choices. Direct ratings and DCE-derived importance produced different attribute rankings, but this difference should not be interpreted as evidence of subconscious decision-making. The subgroup findings were exploratory and did not establish formal differences by clinical department or years of professional experience. Because the study assessed stated preferences rather than actual prescribing behavior or clinical outcomes, the results should be viewed as informing the design of evidence summaries, decision-support tools, and structured communication approaches, whose effectiveness and safety impact require prospective evaluation.

## Data Availability

The original contributions presented in the study are included in the article/Supplementary Material, further inquiries can be directed to the corresponding author.
