# Supplementary material for: Physician preferences for prescribing Chinese patent medicines during pregnancy: a discrete choice experiment
Source: Front Pharmacol. 2026 Aug 3;17:1880942. doi: 10.3389/fphar.2026.1880942 (PMC13478104; doi:10.3389/fphar.2026.1880942)
Supplement: Supplementary file 1 [file Supplementaryfile1.docx]

**Supplementary Material. Variable coding and Stata command structure for the mixed logit model**

**1 Data structure**

The DCE dataset was organized in long format, with each row representing one alternative within one choice task. Each respondent completed 12 experimental choice tasks, and each task included two alternatives. Therefore, each respondent contributed 24 alternative-level rows to the model estimation.

The key variables were as follows:

**person_id**: respondent identifier.

**grip**: unique choice-set identifier across all respondents and choice tasks.

**set**: choice-task number within each questionnaire version, ranging from 1 to 12.

**alt_id**: alternative identifier within each choice task.

**select**: dependent variable, coded as 1 if the alternative was selected and 0 otherwise.

**2 Raw attribute coding**

The raw categorical attributes were initially coded as follows:

**influence**: fetal safety impact; 1 = no fetal impact, 2 = minor fetal impact, 3 = significant fetal impact.

**stage**: treatment stage; 1 = early pregnancy, 2 = mid-pregnancy, 3 = late pregnancy.

**willingness**: patient willingness; 1 = willing, 0 = unwilling.

**experience**: prior prescribing experience with the specific CPM; 1 = experienced, 0 = not experienced.

**route**: route of administration; 1 = oral, 2 = topical, 3 = other route.

**economic_level**: patient affordability; 1 = affordable, 0 = unaffordable.

**effect**: drug efficacy; 1 = uncertain, 2 = average, 3 = good.

Before model estimation, categorical attributes were transformed into dummy variables. The reference levels were significant fetal impact, late pregnancy, unwillingness, no prior prescribing experience, other route of administration, unaffordability, and good efficacy.

**3 Dummy-variable generation**

* Fetal safety impact; reference = significant fetal impact
gen no_impact = (influence == 1) if !missing(influence)
gen minor_impact = (influence == 2) if !missing(influence)

* Treatment stage; reference = late pregnancy
gen early_pregnancy = (stage == 1) if !missing(stage)
gen mid_pregnancy = (stage == 2) if !missing(stage)

* Patient willingness; reference = unwilling
gen willing = (willingness == 1) if !missing(willingness)

* Prior prescribing experience with the specific CPM; reference = not experienced
gen experienced = (experience == 1) if !missing(experience)

* Route of administration; reference = other route
gen oral = (route == 1) if !missing(route)
gen topical = (route == 2) if !missing(route)

* Patient affordability; reference = unaffordable
gen affordable = (economic_level == 1) if !missing(economic_level)

* Drug efficacy; reference = good
gen uncertain_efficacy = (effect == 1) if !missing(effect)
gen average_efficacy = (effect == 2) if !missing(effect)

**4 Conditional logit model**

A conditional logit model was first estimated for model comparison. Standard errors were clustered at the respondent level to account for repeated choices by the same physician; this clustering does not change the likelihood-based AIC or BIC used for model comparison.

```
clogit select minor_impact no_impact ///
    mid_pregnancy early_pregnancy ///
    willing experienced ///
    topical oral ///
    affordable ///
    average_efficacy uncertain_efficacy, ///
    group(grip) vce(cluster person_id)

estimates store clogit_model
```

**5 Mixed logit model**

The final mixed logit model was estimated using the user-written mixlogit command in Stata 16.0. All non-reference attribute-level coefficients were specified as normally distributed random parameters. The model was estimated using 500 simulated draws.

```
capture which mixlogit
if _rc ssc install mixlogit

set seed 12345
mixlogit select minor_impact no_impact ///
    mid_pregnancy early_pregnancy ///
    willing experienced ///
    topical oral ///
    affordable ///
    average_efficacy uncertain_efficacy, ///
    group(grip) id(person_id) ///
    rand(minor_impact no_impact ///
         mid_pregnancy early_pregnancy ///
         willing experienced ///
         topical oral ///
         affordable ///
         average_efficacy uncertain_efficacy) ///
    nrep(500)

estimates store mixlogit_model
```

The group(grip) option identified the choice set, and the id(person_id) option identified repeated choices made by the same respondent. The rand() option specified the variables treated as random parameters, and nrep(500) specified 500 simulated draws.

**6 Model comparison**

estimates stats clogit_model mixlogit_model

Model fit was compared using the Akaike information criterion and Bayesian information criterion. The mixed logit model was selected as the final model because it had lower AIC and BIC values.

**7 Random-parameter standard deviations**

estimates restore mixlogit_model
mixlcov, sd

The mixlcov, sd command was used to display the estimated standard deviations of the random parameters.

**8 Exploratory subgroup models**

Subgroup analyses were conducted using the same model specification. These models were estimated separately and were interpreted descriptively because no formal interaction tests were performed. The clinical-department variable was coded as 1 = Department of Traditional Chinese Medicine and 2 = Department of Obstetrics and Gynecology. The experience-group variable was coded as 0 = 10 years or less and 1 = more than 10 years of professional experience.

* Clinical department and experience-group labels
label define clinical_department_lbl 1 "Department of Traditional Chinese Medicine" 2 "Department of Obstetrics and Gynecology"
label values clinical_department clinical_department_lbl

label define exp_group_lbl 0 "<=10 years" 1 ">10 years"
label values exp_group exp_group_lbl

**8.1 Clinical department**

```
* Department of Traditional Chinese Medicine
mixlogit select minor_impact no_impact ///
    mid_pregnancy early_pregnancy ///
    willing experienced ///
    topical oral ///
    affordable ///
    average_efficacy uncertain_efficacy ///
    if clinical_department == 1, ///
    group(grip) id(person_id) ///
    rand(minor_impact no_impact ///
         mid_pregnancy early_pregnancy ///
         willing experienced ///
         topical oral ///
         affordable ///
         average_efficacy uncertain_efficacy) ///
    nrep(500)

estimates store mix_tcm

* Department of Obstetrics and Gynecology
mixlogit select minor_impact no_impact ///
    mid_pregnancy early_pregnancy ///
    willing experienced ///
    topical oral ///
    affordable ///
    average_efficacy uncertain_efficacy ///
    if clinical_department == 2, ///
    group(grip) id(person_id) ///
    rand(minor_impact no_impact ///
         mid_pregnancy early_pregnancy ///
         willing experienced ///
         topical oral ///
         affordable ///
         average_efficacy uncertain_efficacy) ///
    nrep(500)

estimates store mix_obgyn
```

**8.2 Years of professional experience**

```
* 10 years or less of professional experience
mixlogit select minor_impact no_impact ///
    mid_pregnancy early_pregnancy ///
    willing experienced ///
    topical oral ///
    affordable ///
    average_efficacy uncertain_efficacy ///
    if exp_group == 0, ///
    group(grip) id(person_id) ///
    rand(minor_impact no_impact ///
         mid_pregnancy early_pregnancy ///
         willing experienced ///
         topical oral ///
         affordable ///
         average_efficacy uncertain_efficacy) ///
    nrep(500)

estimates store mix_exp_le10

* More than 10 years of professional experience
mixlogit select minor_impact no_impact ///
    mid_pregnancy early_pregnancy ///
    willing experienced ///
    topical oral ///
    affordable ///
    average_efficacy uncertain_efficacy ///
    if exp_group == 1, ///
    group(grip) id(person_id) ///
    rand(minor_impact no_impact ///
         mid_pregnancy early_pregnancy ///
         willing experienced ///
         topical oral ///
         affordable ///
         average_efficacy uncertain_efficacy) ///
    nrep(500)

estimates store mix_exp_gt10
```
